# Supplementary material for: Invasive Streptococcal Infection Can Lead to the Generation of Cross-Strain Opsonic Antibodies
Source: Microbiol Spectr. 2022 Oct 31;10(6):e02486-22. doi: 10.1128/spectrum.02486-22 (PMC9769875; doi:10.1128/spectrum.02486-22)
Supplement: Supplemental file 1 — Supplemental material. Download spectrum.02486-22-s0001.pdf, PDF file, 1.9 MB [file spectrum.02486-22-s0001.pdf]

Supplement Figure 1

A

Antibody Affinity range

PA

Xolair	K_DFc	419.5	± 1875.9 nM^{-1}
	log_{10} reference nM^{-1}		
Acute	K_D Mean	62.2	± 5.4
	K_D Range	0.0	± 16.0
Convalescent	K_D Mean	59.2	± 4.7
	K_D Range	0.0	± 21.6
IVIG	K_D Mean	58.4	± 2.4
	K_D Range	0.0	± 0.0

PB

Xolair	K_DFc	-5.9	± 3.6 nM^{-1}
	log_{10} reference nM^{-1}		
Acute	K_D Mean	93.5	± 4.7
	K_D Range	0.0	± 0.0
Convalescent	K_D Mean	93.2	± 4.6
	K_D Range	0.0	± 0.0
IVIG	K_D Mean	82.9	± 1240.2
	K_D Range	0.0	± 2507.8

PC

Xolair	K_DFc	-2.0	± 3.8 nM^{-1}
	log_{10} reference nM^{-1}		
Acute	K_D Mean	107.0	± 2.6
	K_D Range	0.0	± 0.0
Convalescent	K_D Mean	84.3	± 4.4
	K_D Range	0.0	± 0.0
IVIG	K_D Mean	85.8	± 5.7
	K_D Range	0.0	± 0.0

PD

Xolair	K_DFc	119.7	± 55.8 nM^{-1}
	log_{10} reference nM^{-1}		
Acute	K_D Mean	58.6	± 7.3
	K_D Range	0.0	± 0.0
Convalescent	K_D Mean	57.2	± 6.7
	K_D Range	0.0	± 0.0
IVIG	K_D Mean	52.5	± 5.5
	K_D Range	0.0	± 0.0

Supplementary Figure 1.

(A) Quantifying the affinities of each serum to M-protein through modeling of binding data. The affinity for sera and IVIG expressed in K_D mean log10 reference nM^{-1} while for Xolair, the M-protein Fc affinity is determined, named K_DFc with nM^{-1} as the unit.

Supplement Figure 2

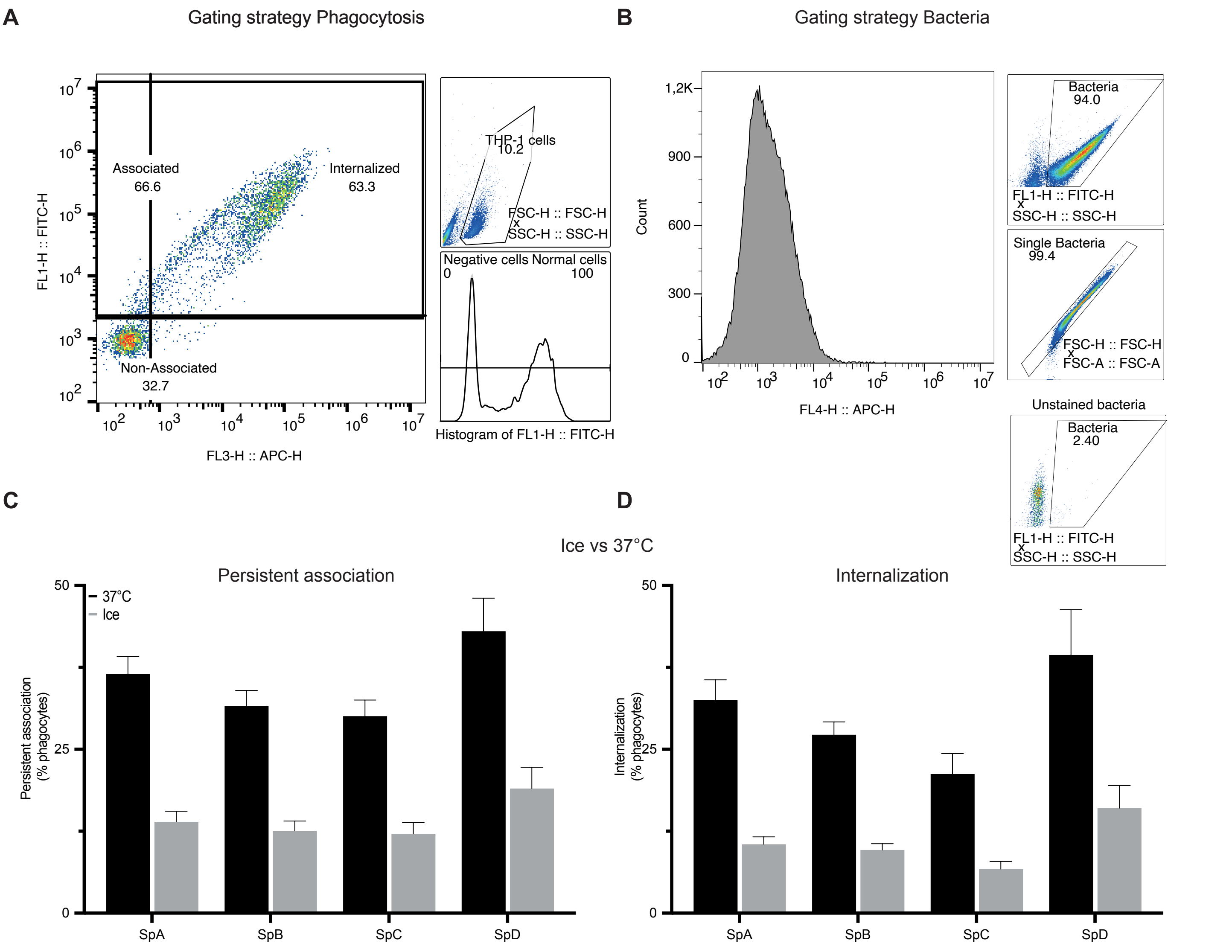

Supplementary Figure 2.
(A-B) Gating strategy of flow cytometry data for phagocytes (A) and bacteria (B) using FlowJo. (C-D) The clinical *S. pyogenes* isolates (Sp) opsonized with 0.5 mg/ml IVIG with phagocytosis (C: association, D: internalization) either on ice or 37°C. Data were acquired through flow cytometry and are presented as mean ±SD, n = 4. .

Supplement Figure 3

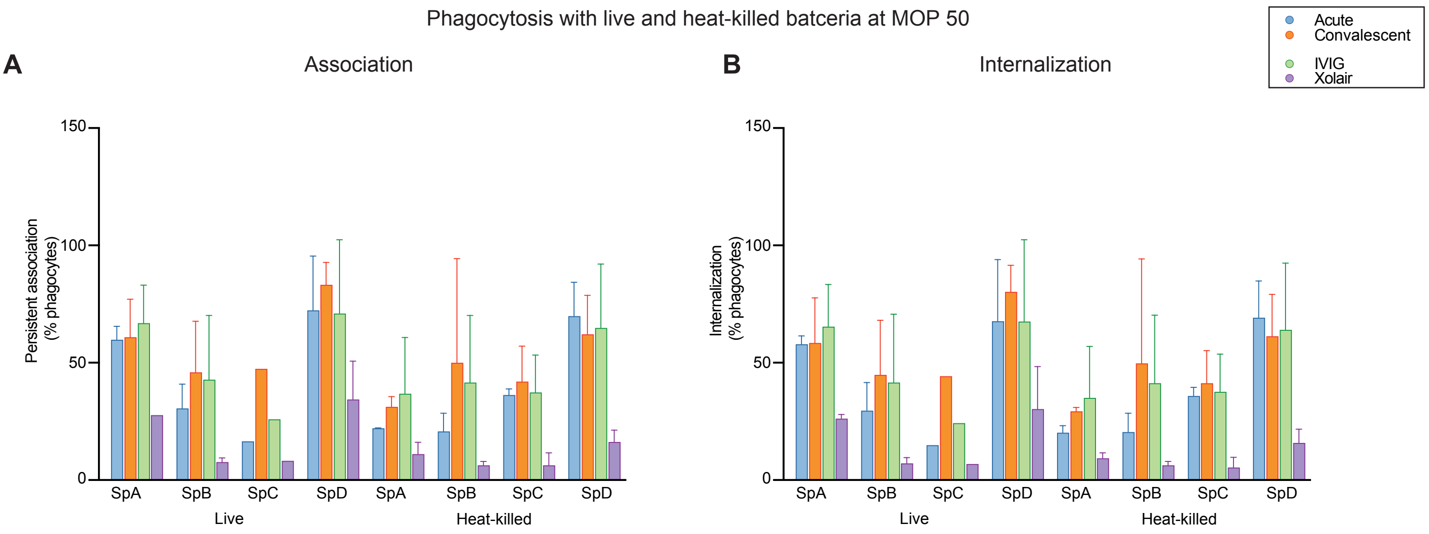

Supplementary Figure 3.
(A-B) The comparison of phagocytosis between live and heat-killed bacteria isolates. The acute (blue) and convalescent (orange) sera from four patients (PA-PD) with *S. pyogenes* invasive infection were assessed. The different isolates (Sp) were cultured to log phase, heat-killed or kept alive on ice before fluorescently doubled stained with a pH stable (Oregon Green) and a pH-sensitive (CypHer-5E) dye and opsonized in 5 % of corresponding serum. The concentration was 0.5 mg/ml for polyclonal IgG (IVIG, green) and monoclonal non-specific IgG (Xolair, purple). Phagocytosis was performed with THP-1 cells incubated (30 min, 150 µl, MOP 50, 37°C) with each isolate. (A) Visualizes % phagocytes associating with bacterium and (B) % phagocytes internalizing bacterium. Data were acquired through flow cytometry and are presented as mean ±SD, n = 2 (except SpC live n=1).

Supplement Figure 4

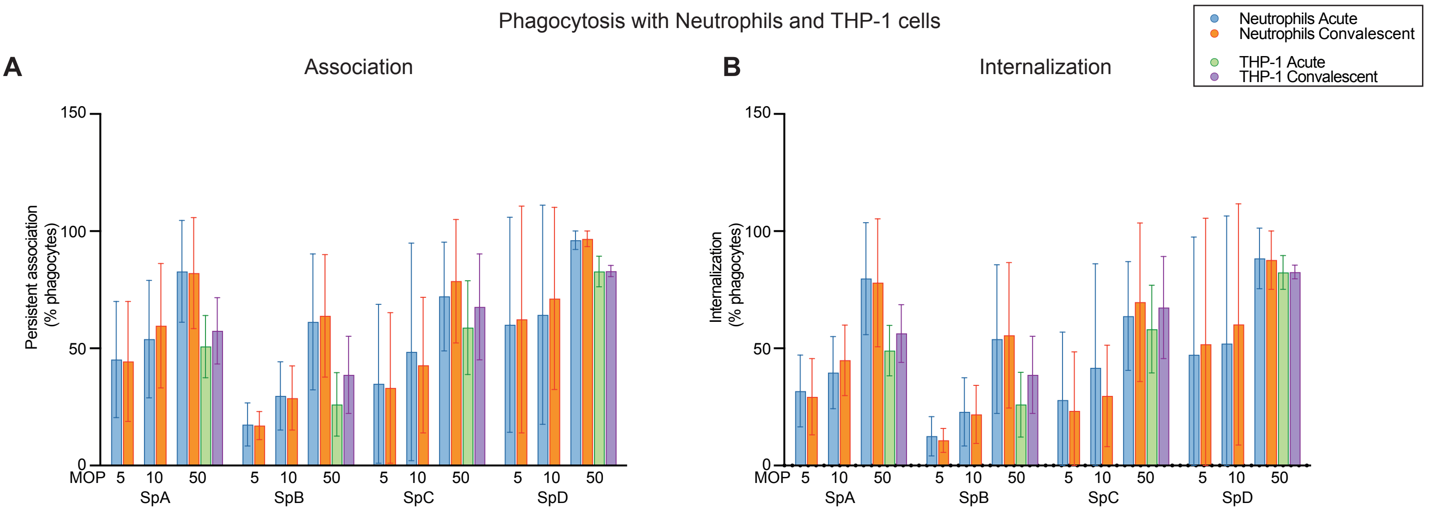

Supplementary Figure 4.
(A-B) A comparison of phagocytosis between neutrophils and THP-1 cells with heat-killed bacteria isolates. The acute (blue and green) and convalescent (orange and purple) sera from four patients (PA-PD) with *S. pyogenes* invasive infection were used. The different isolates (Sp) were fluorescently doubled stained with a pH stable (Oregon Green) and a pH-sensitive (CypHer-5E) dye and opsonized in 5 % of corresponding serum. Neutrophils were isolated from healthy-donors. Phagocytosis was performed with neutrophils (blue and orange) or THP-1 cells (green and purple) incubated (37°C, 30 min, 150 µl, MOP5-50 for neutrophils and MOP 50 for THP-1 cells) with each isolate. (A) Visualizes % phagocytes associating with bacterium and (B) % phagocytes internalizing bacterium. Data were acquired through flow cytometry and are presented as mean ±SD, n = 2.

Supplement Figure 5

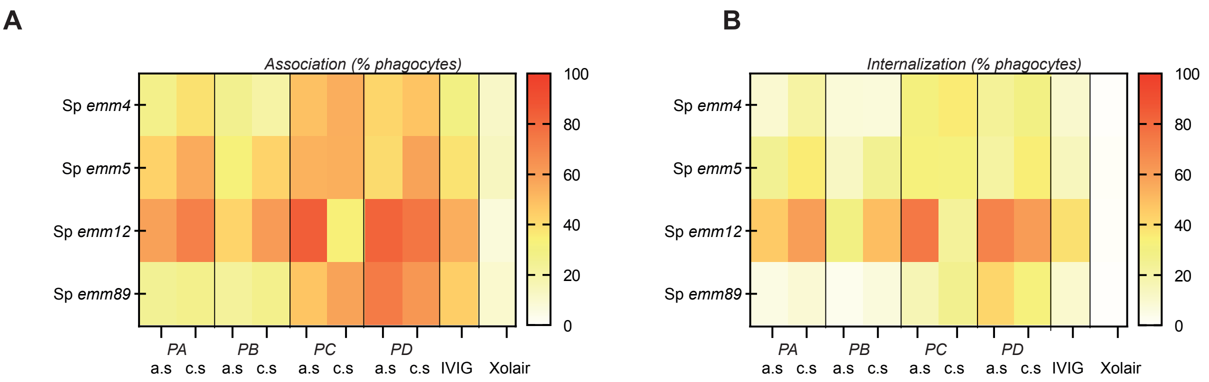

Supplementary Figure 5. (A-B) Phagocytosis assessment of other bacteria isolates with the acute (a.s) and convalescent (c.s) sera from four patients (PA-PD) with *S. pyogenes* invasive infection. Four *S. pyogenes* (Sp) isolates with other *emm*-type (*emm4, emm5, emm12, emm89*) were heat-killed and fluorescently doubled stained with a pH stable (Oregon Green) and a pH-sensitive (CypHer-5E) dye and opsonized in 5 % of each serum. The concentration was 0.5 mg/ml for polyclonal IgG (IVIG) and monoclonal non-specific IgG (Xolair). Phagocytosis was performed with THP-1 cells incubated (30 min, 150 µl, MOP 50, 37°C) with each isolate. Data were acquired through flow cytometry and are presented as mean, n = 2 (except Sp *emm89* c.s, n=1).

Supplementary Data. M-protein sequences

```
########################################
# Program: needle
# Rundate: Sun  9 Jan 2022 20:26:26
# Commandline: needle
#    -auto
#    -stdout
#    -asequence emboss_needle-I20220109-203443-0851-42254984-p1m.asequence
#    -bsequence emboss_needle-I20220109-203443-0851-42254984-p1m.bsequence
#    -datafile EBLOSUM62
#    -gapopen 10.0
#    -gapextend 0.5
#    -endopen 10.0
#    -endextend 0.5
#    -aformat3 pair
#    -sprotein1
#    -sprotein2
# Align_format: pair
# Report_file: stdout
########################################
#=======================================
# Aligned_sequences: 2
# 1: M1-SPB-4
# 2: M85-SPD
# Matrix: EBLOSUM62
# Gap_penalty: 10.0
# Extend_penalty: 0.5
#
# Length: 443
# Identity:     136/443 (30.7%)
# Similarity:   187/443 (42.2%)
# Gaps:         178/443 (40.2%)
# Score: 529.0
#=======================================

M1-SPB-4           1 MAKNNTNRHYSLRKLKTGTASVAVALTVLGAGFANQTEVKANGDGNPREV     50
                     |.:.:||:.||||||||||||||||:.||||||||||||||.....|:..
M85-SPD            1 MVRKDTNKQYSLRKLKTGTASVAVAVAVLGAGFANQTEVKAAEQRAPQAK     50

M1-SPB-4          51 IEDLAANNPAIQNIRLRHENKDLKARLENAMEVAGRDFKRAEELEKAKQA    100
                     ...::|:   :.| .|..|||.|:       |..|....:.:..|...:.
M85-SPD           51 GTSVSAD---LYN-SLWDENKTLR-------EKQGEYITKIQNEETKNKE     89

M1-SPB-4         101 LEDQRKDLETKLKELQQDYDLAKESTSWDRQRLEKELEEKKEALELAIDQ    150
                     |:.:.|:|::::.:|   .|:.:..        ::|||.|:...|..:.|
M85-SPD           90 LDKKNKELDSRVTDL---IDVIEHD--------DQELERKERMYEAFLKQ    128

M1-SPB-4         151 ASRDYHRATALEKELEEKKALELAIDQASQDYNRANVLEKELETITREQ    200
                     :....:..||.:..|.||.|.||               .:|::...:| :
M85-SPD          129 SKDQVNNLTAEKDTLAEKAKKLE---------------EDKQISDASR-K    162

M1-SPB-4         201 EINRNLLGN--AKLELDQLSSEKEQLTIEKAKLEEEKQISDASRQSLRRD    248
                     .::|:|.|:  ||.||:   ::.::|..|..||:|:|||||||||.|.||
M85-SPD          163 SLSRDLEGSRAAKKELE---AKHQKLETEHQKLKEDKQISDASRQGLSRD    209

M1-SPB-4         249 LDASREAKKQVEKDLANLTAELDKVKEEKQISDASRQGLRRDLDASREAK    298
                     |:|||||||:||.|||.||||..|:||||||||||||||.|||:||||||
M85-SPD          210 LEASREAKKKVEADLAALTAEHQKLKEEKQISDASRQGLSRDLEASREAK    259

M1-SPB-4         299 KQVEKDLA------------------------------------------    306
                     |:||.|||
M85-SPD          260 KKVEADLAEANSKLQALEKLNKELEEGKKLSEKEKAELQARLEAEAKALK    309

M1-SPB-4         307 --------------------------------------------------    306

M85-SPD          310 EQLAKQAEELAKLRAGKASDSQTPDAKPGNKVVPGKGQAPQAGTKPNQNK    359

M1-SPB-4         307 -------------------------------------------    306

M85-SPD          360 APMKETKRQLPSTGEAANPFFTAAAATVMVSAGMLALKRKEEN    402
#---------------------------------------
#---------------------------------------
```

```
########################################
# Program: needle
# Rundate: Sun  9 Jan 2022 20:27:19
# Commandline: needle
#    -auto
#    -stdout
#    -asequence emboss_needle-I20220109-203901-0362-16640114-p1m.asequence
#    -bsequence emboss_needle-I20220109-203901-0362-16640114-p1m.bsequence
#    -datafile EBLOSUM62
#    -gapopen 10.0
#    -gapextend 0.5
#    -endopen 10.0
#    -endextend 0.5
#    -aformat3 pair
#    -sprotein1
#    -sprotein2
# Align_format: pair
# Report_file: stdout
########################################

#=======================================
# Aligned_sequences: 2
# 1: M1-SPB-4
# 2: M118-SPA
# Matrix: EBLOSUM62
# Gap_penalty: 10.0
# Extend_penalty: 0.5
#
# Length: 422
# Identity:     121/422 (28.7%)
# Similarity:   162/422 (38.4%)
# Gaps:         206/422 (48.8%)
# Score: 447.5
#=======================================

M1-SPB-4           1 MAKNNTNRHYSLRKLKTGTASVAVALTVLGAGFANQTEVKANGDGNPREV     50
                     ||:.:||:.||||||||||||||||:.||||||||||||||
M118-SPA           1 MARKDTNKQYSLRKLKTGTASVAVAVAVLGAGFANQTEVKA---------     41

M1-SPB-4          51 IEDLAANNPAIQNIRLRHENKDLKARLENAMEVAGRDFKRAEELEKAKQA    100
                              |.:.:.:...|....|:|.|  ::|....|..|.||:.::
M118-SPA          42 ---------AEKKVEVADSNASSVAKLYN--QIADLTDKNGEYLERIEE-     79

M1-SPB-4         101 LEDQRKDLETKLKELQQDYDLAKESTSWDRQRLEKELEEKKEALELAIDQ    150
                     ||:::|:||                      :||::            .|
M118-SPA          80 LEERQKNLE----------------------KLERQ------------SQ     95

M1-SPB-4         151 ASRDYHRATALEKELEEKKKALELAIDQASQDYNRANVLEKELETITREQ    200
                     .:.|.|....::|..|.|::..|               .:|.||.:.|:.
M118-SPA          96 VAADKHYQEQVKKHQEYKQEQEE---------------RQKNLEELERQN    130

M1-SPB-4         201 EINRNLLGNAKLELDQLSSEKEQLTIEKAKLEEEKQISDASRQSLRRDLD    250
                               |.|:|:...|:.|   ::.:||.|||||:|||:||.|||:
M118-SPA         131 ----------KREIDKRYQEQLQ---KQQQLETEKQISEASRKSLSRDLE    167

M1-SPB-4         251 ASREAKKQVEKDLANLTAELDKVKEEKQISDASRQGLRRDLDASREAKKQ    300
                     |||.|||.:|       ||..|:||||||||||||||.|||:|||||||:
M118-SPA         168 ASRAAKKDLE-------AEHQKLKEEKQISDASRQGLSRDLEASREAKKK    210

M1-SPB-4         301 VEKDLA--------------------------------------------    306
                     ||.|||
M118-SPA         211 VEADLAEANSKLQALEKLNKELEEGKKLSEKEKAELQAKLEAEAKALKEQ    260

M1-SPB-4         307 --------------------------------------------------    306

M118-SPA         261 LAKQAEELAKLKGNQTPNAKVAPQANRSRSAMTQQKRTLPSTGETANPFF    310

M1-SPB-4         307 ----------------------    306

M118-SPA         311 TAAAATVMVSAGMLALKRKEEN    332

#---------------------------------------
#---------------------------------------
```

```
########################################
# Program: needle
# Rundate: Sun  9 Jan 2022 20:41:42
# Commandline: needle
#    -auto
#    -stdout
#    -asequence emboss_needle-I20220109-204141-0310-42199984-p2m.asequence
#    -bsequence emboss_needle-I20220109-204141-0310-42199984-p2m.bsequence
#    -datafile EBLOSUM62
#    -gapopen 10.0
#    -gapextend 0.5
#    -endopen 10.0
#    -endextend 0.5
#    -aformat3 pair
#    -sprotein1
#    -sprotein2
# Align_format: pair
# Report_file: stdout
########################################

#=======================================
#
# Aligned_sequences: 2
# 1: M85-SPD
# 2: M118-SPA
# Matrix: EBLOSUM62
# Gap_penalty: 10.0
# Extend_penalty: 0.5
#
# Length: 403
# Identity:     239/403 (59.3%)
# Similarity:   281/403 (69.7%)
# Gaps:          72/403 (17.9%)
# Score: 1016.5
# 
#
#=======================================

M85-SPD            1 MVRKDTNKQYSLRKLKTGTASVAVAVAVLGAGFANQTEVKAAEQRAPQAK     50
                     |.|||||||||||||||||||||||||||||||||||||||||::...|.
M118-SPA           1 MARKDTNKQYSLRKLKTGTASVAVAVAVLGAGFANQTEVKAAEKKVEVAD     50

M85-SPD           51 GTSVS-ADLYNSLWDENKTLREKQGEYITKIQNEETKNKELDKKNKELDS     99
                     ..:.| |.|||.:.|    |.:|.|||:.:|:..|.:.|.|:|       
M118-SPA          51 SNASSVAKLYNQIAD----LTDKNGEYLERIEELEERQKNLEK-------     89

M85-SPD          100 RVTDLIDVIEHDDQELERKERMYEAFLKQSKDQVNNLTAEKDTLAEKAKK    149
                                    |||:               :.:.|:|. ..|:.||
M118-SPA          90 ---------------LERQ---------------SQVAADKH-YQEQVKK    108

M85-SPD          150 LEEDKQISDASRKSLSRDLEGSRAAKKELEAKHQKLETEHQKLKEDKQIS    199
                     .:|.||..:..:|:| .:||  |..|:|::.::|:...:.|:|:.:||||
M118-SPA         109 HQEYKQEQEERQKNL-EELE--RQNKREIDKRYQEQLQKQQQLETEKQIS    155

M85-SPD          200 DASRQGLSRDLEASREAKKKVEADLAALTAEHQKLKEEKQISDASRQGLS    249
                     :|||:.|||||||||.|||.:|       |||||||||||||||||||||
M118-SPA         156 EASRKSLSRDLEASRAAKKDLE-------AEHQKLKEEKQISDASRQGLS    198

M85-SPD          250 RDLEASREAKKKVEADLAEANSKLQALEKLNKELEEGKKLSEKEKAELQA    299
                     ||||||||||||||||||||||||||||||||||||||||||||||||||
M118-SPA         199 RDLEASREAKKKVEADLAEANSKLQALEKLNKELEEGKKLSEKEKAELQA    248

M85-SPD          300 RLEAEAKALKEQLAKQAEELAKLRAGKASDSQTPDAKPGNKVVPGKGQAP    349
                     :||||||||||||||||||||||:.     :|||:||          .||
M118-SPA         249 KLEAEAKALKEQLAKQAEELAKLKG-----NQTPNAK----------VAP    283

M85-SPD          350 QAGTKPNQNKAPMKETKRQLPSTGEAANPFFTAAAATVMVSAGMLALKRK    399
                     ||    |::::.|.:.||.||||||.||||||||||||||||||||||||
M118-SPA         284 QA----NRSRSAMTQQKRTLPSTGETANPFFTAAAATVMVSAGMLALKRK    329

M85-SPD          400 EEN    402
                     |||
M118-SPA         330 EEN    332

#---------------------------------------
#---------------------------------------
```

CLUSTAL O(1.2.4) multiple sequence alignment

```
M1-SPB-4      MAKNNTNRHYSLRKLKTGTASVAVALTVLGAGFANQTEVKANGDGNPREVIEDLAANNPA 60
M85-SPD       MVRKDTNKQYSLRKLKTGTASVAVAVAVLGAGFANQTEVKAAEQRAPQAKGTSVSADL-- 58
M118-SPA      MARKDTNKQYSLRKLKTGTASVAVAVAVLGAGFANQTEVKAAEKKVEVADSNAS---S-- 55
              *.:::**::****************::**************  .

M1-SPB-4      IQNIRLRHENKDLKARLENAMEVAGRDFKRAEELEKAKQALEDQRKDLETKLKELQQDYD 120
M85-SPD       --YNSLWDENKTLREK-------QGEYITKIQNEETKNKELDKKNKELDSRVTDLIDV-- 107
M118-SPA      --VAKLYNQIADLTDK-------NGEYLERIEELEERQKNLEKLERQSQVAA-------- 98
                   * .:   *  :        *. : : :: *  :: *:. .:: :

M1-SPB-4      LAKESTSWDRQRLEKELEEKKEALELAIDQASRDYHRATALEKELEEKKALELAIDQAS 180
M85-SPD       -----IEHDDQEL----ERKERMYEAFLKQSKDQVNNLTAEKDTLAEKAKKLEED--KQI 156
M118-SPA      --------------------DKHYQEQVKKHQE-------YKQEQEERQKNLEE------ 125
                                  ..  :  :.: .         :.   *: * **

M1-SPB-4      QDYNRANVLEKELETITREQEINRNLLGNAKLELDQLSSEKEQLTIEKAKLEEEKQISDA 240
M85-SPD       SDAS--------------RKSLSRDLEGS-RAAKKELEAKHQKLETEHQKLKEDKQISDA 201
M118-SPA      ---------------------------LE-RQNKREIDKRYQEQLQKQQLETEKQISEA 157
                                          . :    ::. . ::   :: :*: :****:*

M1-SPB-4      SRQSLRRDLDASREAKKQVEKDLANLTAELDKVKEEKQISDASRQGLRRDLDASREAKKQ 300
M85-SPD       SRQGLSRDLEASREAKKKVEADLAALTAEHQKLKEEKQISDASRQGLSRDLEASREAKKK 261
M118-SPA      SRKSLSRDLEASRAAKKD-------LEAEHQKLKEEKQISDASRQGLSRDLEASREAKKK 210
              **:.* ***:*** ***.       * ** :*:************** ***:*******:

M1-SPB-4      VEKDLA------------------------------------------------------ 306
M85-SPD       VEADLAEANSKLQALEKLNKELEEGKKLSEKEKAELQARLEAEAKALKEQLAKQAEELAK 321
M118-SPA      VEADLAEANSKLQALEKLNKELEEGKKLSEKEKAELQAKLEAEAKALKEQLAKQAEELAK 270
              ** ***

M1-SPB-4      ------------------------------------------------------------ 306
M85-SPD       LRAGKASDSQTPDAKPGNKVVPGKGQAPQAGTKPNQNKAPMKETKRQLPSTGEAANPFFT 381
M118-SPA      LKGNQTPN---------AKVA----------PQANRSRSAMTQQKRTLPSTGETANPFFT 311


M1-SPB-4      ---------------------  306
M85-SPD       AAAATVMVSAGMLALKRKEEN  402
M118-SPA      AAAATVMVSAGMLALKRKEEN  332
```
